# Communities catalyzing change with data to mitigate an invisible menace, traffic-related air pollution

**DOI:** 10.1186/s12889-024-17864-9

**Published:** 2024-02-08

**Authors:** Linda Sprague Martinez, Shir Lerman Ginzburg, Sharon Ron, Cristina Araujo Brinkerhoff, Samiya Haque, Sophia Angali England, Kynza Khimani, Wig Zamore, Ellin Reisner, Lydia Lowe, Doug Brugge

**Affiliations:** 1 School of Social Work, Macro Department, Boston University, Boston, MA USA; 2 Department of Public Health, MCPHS University, Boston, MA USA; 3 Metropolitan Area Planning Council, Boston, MA USA; 4 Somerville Transportation Equity Partnership, Somerville, MA USA; 5 Chinatown Community Land Trust, Boston, MA USA; 6 School of Medicine, Department of Public Health Sciences, University of Connecticut, Farmington, CT USA; 7 School of Medicine, University of Connecticut, Health Disparities Institute, 241 Main Street, Hartford, CT 06106 USA

**Keywords:** Transportation relation air pollution (TRAP), Ultrafine particles, CBPR, Community action, Research translation

## Abstract

**Objectives:**

To identify strategies and tactics communities use to translate research into environmental health action.

**Methods:**

We employed a qualitative case study design to explore public health action conducted by residents, organizers, and public health planners in two Massachusetts communities as part of a community based participatory (CBPR) research study. Data sources included key informant interviews (*n* = 24), reports and direct observation of research and community meetings (*n* = 10) and project meeting minutes from 2016–2021. Data were coded deductively drawing on the community organizing and implementation frameworks.

**Results:**

In Boston Chinatown, partners drew broad participation from community-based organizations, residents, and municipal leaders, which resulted in air pollution mitigation efforts being embedded in the master planning process. In Somerville, partners focused on change at multiple levels, developer behavior, and separate from the funded research, local legislative efforts, and litigation.

**Conclusions:**

CBPR affords communities the ability to environmental health efforts in a way that is locally meaningful, leveraging their respective strengths. External facilitation can support the continuity and sustainment of community led CBPR efforts.

Many aspects of the relationship between public health and the built environment have been well documented [1]. There is a robust literature exploring how urban form impacts health and well-being, which includes a focus on roadways [2], walkways [3], housing [4], green space [5], and indoor air quality in buildings [6]. Schultz and Northbridge’s ecological model of environmental health promotion illustrates the multiple pathways through which the built environment shapes health outcomes and how ideology fuels racialized policies that govern the built environment and influence the social context surrounding it [7]. As such, improving the built environment is critical for both addressing health inequities and improving public health overall [8].

Roadways are a critical element of the built environment that affect health because air pollutants and noise are elevated with high levels of traffic. Traffic related air pollution, commonly referred to as TRAP, is defined as a combination of primary and secondary pollutants emitted from vehicles as well as dust from the road that result in harmful gases and particles is the air [9]. Living close to major roadways is associated with poor respiratory, cardiovascular, and neurological outcomes [10]. The public health costs associated with traffic related air pollution (TRAP) can be measured in both years of life lost and in health care expenditures. For example, Farrukh and Kheris (2021) estimate the traffic related air pollution, inclusive of TRAP, in the United States (US) accounted for $178 million in asthma related expenses in 2010 [11]. It is estimated that nearly 20% of the overall population and 27.4% of people of color in the United States live near a major roadway [12]. Further, low-income populations are concentrated near highways [12], and this number is expected to continue to grow with population shifts and development, thus making TRAP a pressing public health issue.

Despite the public health burden of TRAP, community driven interventions to mitigate exposure are limited in the US. This may be associated with the invisibility of TRAP, which is usually odorless and colorless. TRAP related research and action in the US has been geographically isolated, mainly occurring in California communities [13], New York City [14], and Boston [15]. In California, research and action has resulted in state level advocacy and the enactment of protective measures through legislative action [16]. Meanwhile, in European countries multiple municipal TRAP mitigation strategies focus on the built environment including traffic management [17], ventilation [18–22] and land-use [23] as well as infrastructure approaches [23, 24].

The extent to which TRAP mitigation efforts to date have been driven by residents and community-based organizations is unclear. The benefits of community engagement and community driven interventions have been well documented in other contexts [25]. Community members have a nuanced understanding of both the factors that influence health and ways to intervene [26]. Nonetheless, resident engagement in efforts to address TRAP can pose challenges. Communities contend with multiple socio-political and economic factors, such as affordable housing and gentrification; as a result, specific public health issues such as TRAP, an odorless and colorless threat that manifests in disease years or decades in the future, may not be prioritized [27, 28].

Moreover, even in cases in which people in communities prioritize TRAP, addressing it can challenge organizers because in the US, governance of the built environment varies across jurisdictions and can sit at the intersection of municipal, regional, state, and national policy [28]. This can require navigating policy change at multiple levels. Working in partnership with communities and regional planners, researchers can identify areas in which health threats, such as TRAP, intersect with community priorities and implement relevant solutions [29]. More specifically, strong local scientific evidence can put pressure on the logjam that normally prevents or slows policy and practice responses.

The Community Assessment of Freeway Exposure and Health (CAFEH) is a series of community based participatory research (CBPR) studies that leverage community and academic infrastructure to advance scientific understanding of the health risks of TRAP [29], as well as interventions to mitigate the associated risks. CAFEH emerged as the result of community efforts to improve environmental health and as such community partners are involved in all facets of the science and lead action efforts [30]. The CBPR approach associated with CAFEH has been described in the literature [30]and the partnership has accumulated a robust research evidence base focused on the health effects of ultrafine particles which are elevated near highways and major roadways [31]. All too often the research associated with CBPR partnerships is reported without the community action component. Here we focus specifically on the action side of CBPR. Community research partners and regional public health planners led efforts to translate research findings into public health action in two communities, which was informed by Health Lens Analysis (HLA) described in detail in the results. We report on the systems community partners targeted for change and the strategies they employed. The research methodology is described in detail inclusive of the case context. We then present the results and discuss them in the context of the literature.

## Methods

A qualitative case study design was employed to examine TRAP related community level public health action [32, 33]. The study draws on three data sources: key informant interviews with diverse community stakeholders conducted during the summer of 2021, analysis of project meeting minutes and reports, and direct observation of research and community meetings. All protocols were reviewed by the Boston University Charles River Campus Institutional Review Board, protocol #4434X.

### Case context

In 2016, the CAFEH partnership, which was established in 2008, was funded by the National Institute of Environmental Health Sciences (NIEHS) to study the translation of research to public health action aimed at mitigating the health effects associated with TRAP exposures locally, and the community level factors that influence translation [28]. NIEHS has a unique mechanism that funds research to action (R2A) studies, although our study was not funded under this mechanism we employed the research to action framework. Between 2016 and 2021 researchers conducted exposure studies and community partners in Somerville and Chinatown led efforts to translate the science into public health action. Detailed information about the city of Somerville and the neighborhood of Chinatown in Boston and their proximity to the highway has been previously published [28, 34].

**Fig. 1:**
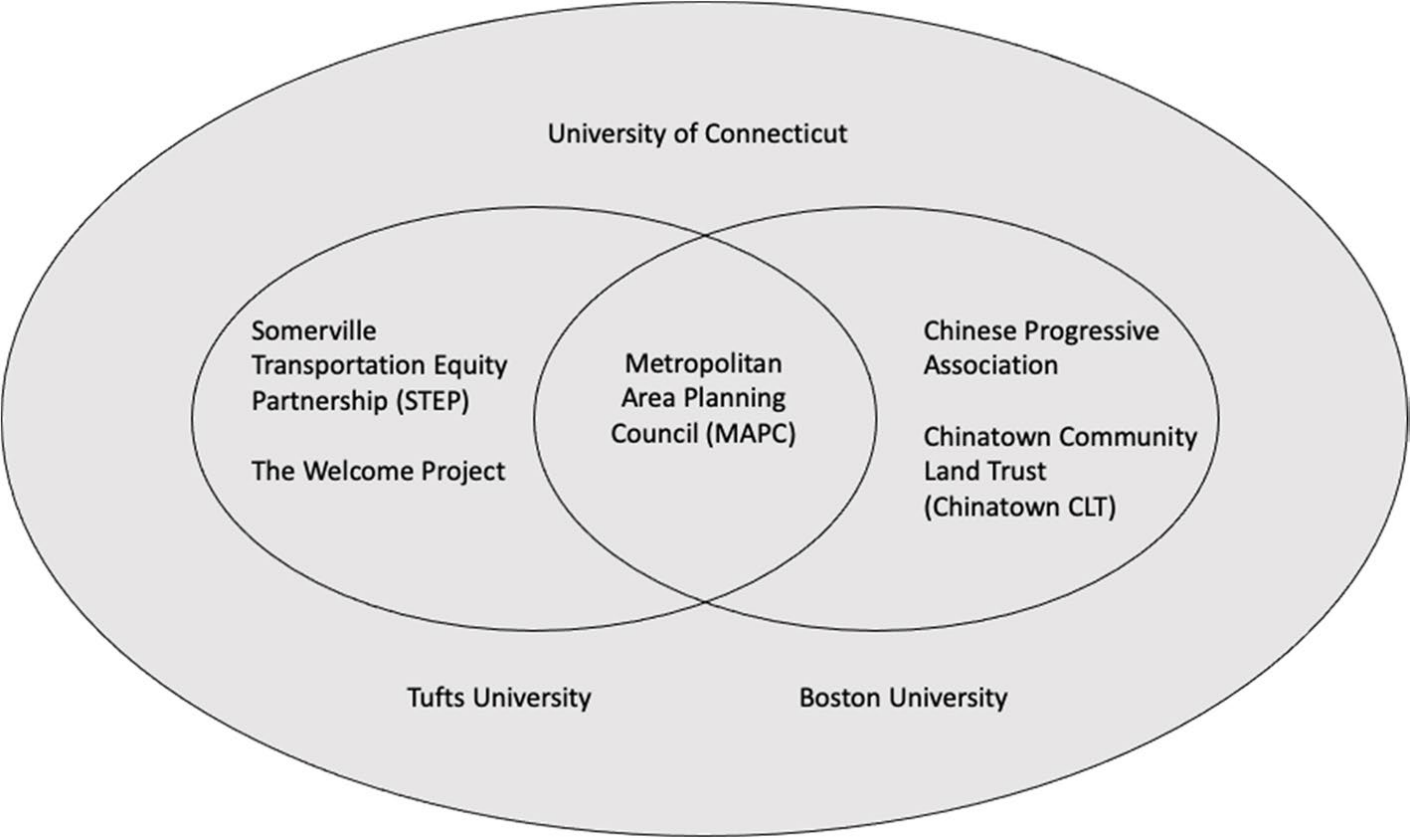
The partnership

The partnership employed a multiple principal investigator structure, led by a university researcher and community leader. University researchers on the study represented three institutions and community research partners were associated with five distinct organizations (see Fig. 1). The study MPIs included an environmental health researcher from the University of Connecticut and an environmental activist from the Somerville Transportation Equity Partnership. Additional Investigators included environmental health scientists from the Tufts University School of Civil and environmental Engineering and Social Work Researchers from Boston University. Non-academic investigators included staff from the Metropolitan Area Planning Council (MAPC), The Welcome Project, The Chinese Progressive Association (CPA), and the Chinatown Community Land Trust (CLT). Public health action efforts were directed by STEP, CPA, CLT and the Welcome Project investigators and logistically coordinated and operationalized with the support of staff from MAPC.

### Data collection methods

A combination of purposive and snowball sampling was used to identify key informants with knowledge of how TRAP related research was being translated into public health action. Informants included team members themselves as well as others in the community with knowledge of the group’s efforts. This included organizational and resident leaders, coalition members, public officials, municipal staff, planners, contractors, organizers, environmental advocates, and environmental lawyers. Attendance records from community meetings were reviewed to generate a preliminary list of potential participants. Meetings were then conducted with partners from each of the communities to generate a list of additional informants. In addition, participants were asked about additional stakeholders that should be contacted. Key informants were contacted by emailed and invited to participate in the study. After each interview participants were asked to identify additional informants.

At the time of the interview, a script outlining the procedures and elements of consent were reviewed and participant questions about the study were addressed. Interviewers then followed a semi-structured interview guide exploring participants’ roles in the community; involvement in efforts to mitigate TRAP; approaches to TRAP related mitigation being employed in the community and challenges to TRAP mitigation. Finally, perceptions of community-level factors influencing research translation and potential challenges and levers were queried. Interviews were audio recorded and transcribed. Transcripts were reviewed to verify their accuracy and then loaded into in NVivo 12.0 qualitative data management software [35].

Observations were conducted from April 2017- May 2021 [28] Observed meetings included bi-weekly research and action planning meetings facilitated by the community principal investigator as well as community meetings held in each community, and design charrettes [28, 36]. Charettes involve a participatory process of engaging diverse community stakeholders in planning, design and problem solving [36, 37]. The overall goal of the observations was to examine dynamics and participation at meetings and public forums associated with the project [36]. Observations were planned in collaboration with the project steering committee. Members of the steering committee introduced the researchers at community meetings and events and explained the goals of the study. Permission was requested to record meetings, when feasible, depending on participants’ comfort with being recorded, the size of the event and the acoustics of the meeting space. At large forums additional note takers were present and introduced to attendees. Comprehensive notes were taken during and at the culmination of each observation [36].

Project documents consulted included protocols, reports, weekly meeting plans, agendas, and committee meeting minutes. The researchers analyzing this data engaged in continuous data collection and analysis and provided feedback to the study steering committee periodically [36].

### Analytic approach

Two types of data analysis were employed: holistic analysis of the whole case and embedded analysis of specific parts of the case [28, 33]. Interviews and observations were coded deductively drawing the on community organizing theory [38, 39]. Two members of the research team coded transcripts using directed content analysis [40]. The data was coded by two independent coders. Content in the text illustrating each code was selected and assigned. Memos were written as coders encountered relevant text that did not fit the coding criteria or in cases where there were questions regarding the text. The researchers met to reconcile codes and memos with a third member of the research team who was engaged to discuss discrepancies and overall themes in the data. During each meeting, intercoder reliability was determined and areas of disagreement were resolved. Directed content analysis allows for emergent themes, in cases where relevant themes emerged that did not fit the coding criteria, an inductive code was developed [40]. After coding was completed, reports were generated using NVivo for each code and summaries were prepared by code [35].

To establish the case, a chronology of events and key themes were examined to assess the complexity of the case [37]. The researchers reviewed interview coding summaries as well as themes from observations and documents multiple times to immerse themselves in the data, to search for meaningful patterns [36, 41] and to reflect on their questions about and reactions to the data [42, 43]. This process was repeated for each of the two communities. The larger story within the data for each community was presented to members of the steering committee, which includes both university and community investigators, to elicit feedback and to further hone the story [28, 33]. Illustrative quotes and examples from documents were then selected to produce a succinct, cogent story of the data within and across the identified themes from the three sources of data [28, 33]. Finally, the research team met to compare and contrast the narratives.

## Results

The case was constructed drawing on 24 key informant interviews. A total of thirty individuals were invited to participate in the interviews (response rate 80%). Of those who did not respond to requests for interviews, four were state and local elected officials and two were community members. Interview participants included public officials, municipal leaders (public health, planning and community development), non-profit leaders, developers, and planners, as well as environmental and social activists and organizers. In addition to interview data, synthesized data were extracted from project reports, meeting observations and steering committee minutes.

As outlined in the partnerships project plan, community partners from Somerville and Chinatown worked in sequential years with a regional planning organization to translate research to public health action. Although the grant outlined communities would use health impact assessments (HIA), early on it became evident to our planning partner that the focus of HIA on a specific, existing policy or project did not align with community partner priorities. As a result, both communities turned to the closely related Health Lens Analysis (HLA) approach to generate and inform planning and policy recommendations to mitigate TRAP [44]. HLA involves an iterative five step process: community engagement, gather evidence, generate solutions and recommendations, navigate implementation, evaluate [44].

In each community, the regional planning partner worked with community research partners to implement the HLA process in an effort to move beyond increasing awareness to advancing community change. They describe their role in the text that following.

*… the HLA process on my end was a lot of trying to get folks around the table within CAFEH but also the larger group of stakeholders that we tapped into while doing that work to define what it is that they wanted to see change.*

Each HLA began with listening sessions during which partners engaged the broader community to explore conceptualizations of health and the social determinants of health as well as health related priority areas within the context of TRAP. Data gathered during listening sessions was paired with additional data collected in each community and used to inform open houses and community design charrettes during which community members engaged in dynamic small group discussions to generate recommendations as well as new viable solutions to mitigate TRAP. Over the course of the HLAs, community partners met independently as well as with the steering community to assess and evaluate recommendations and planned action.

Data indicate that public health action went beyond planning activities conceptualized by the team during the grant writing phase of the project to include individuals from multiple sectors including the arts being integrated into the planning. Although each community began with the same process, they approached the work differently in response to distinct local priorities and contexts. However, similarities were also noted. For example, both communities employed collaborative efforts that involved outreach and education for residents, municipal and state leaders and developers. A brief description of each communities’ HLA is followed by a description of community level engagement and intervention strategies, both within the research project and following upon it on the independent initiative of the community partners.

### Somerville HLA

In Somerville, listening sessions explored community ideas related to noise barriers along the highway [44]. Noise barriers emerged as a priority area as the result of historical inequities during highway construction that left the much of the Interstate-93 in Somerville open, whilst barriers were placed along one side partially shielding the wealthier white population from the highway. Although sound barriers do not eliminate TRAP, they can divert and disburse it if conditions are favorable. As such, community partners felt the long discussed, and never delivered sound barriers, were something around which the community could build support. These discussions were an effective strategy for engaging residents as well as state officials in listening sessions early in the HLA process. Moreover, as described by a planner in the following quote, the topic kept participants involved in planning over time, allowing partners to engage in deeper discussions about other TRAP mitigation strategies.

*…quite early in the process [Somerville]settled around these ideas of noise barriers, and I do think that there are a lot of folks in the group who quite genuinely wanted to build noise barriers. But I think it was also a community planning process to raise this as an issue with the community to talk about it a little bit more holistically and not just say air quality is an issue but talk about what are the impacts on your life and living near a highway and what are some of the mitigation strategies that you would like to see …to improve your sense of well-being.*

**Fig. 2:**
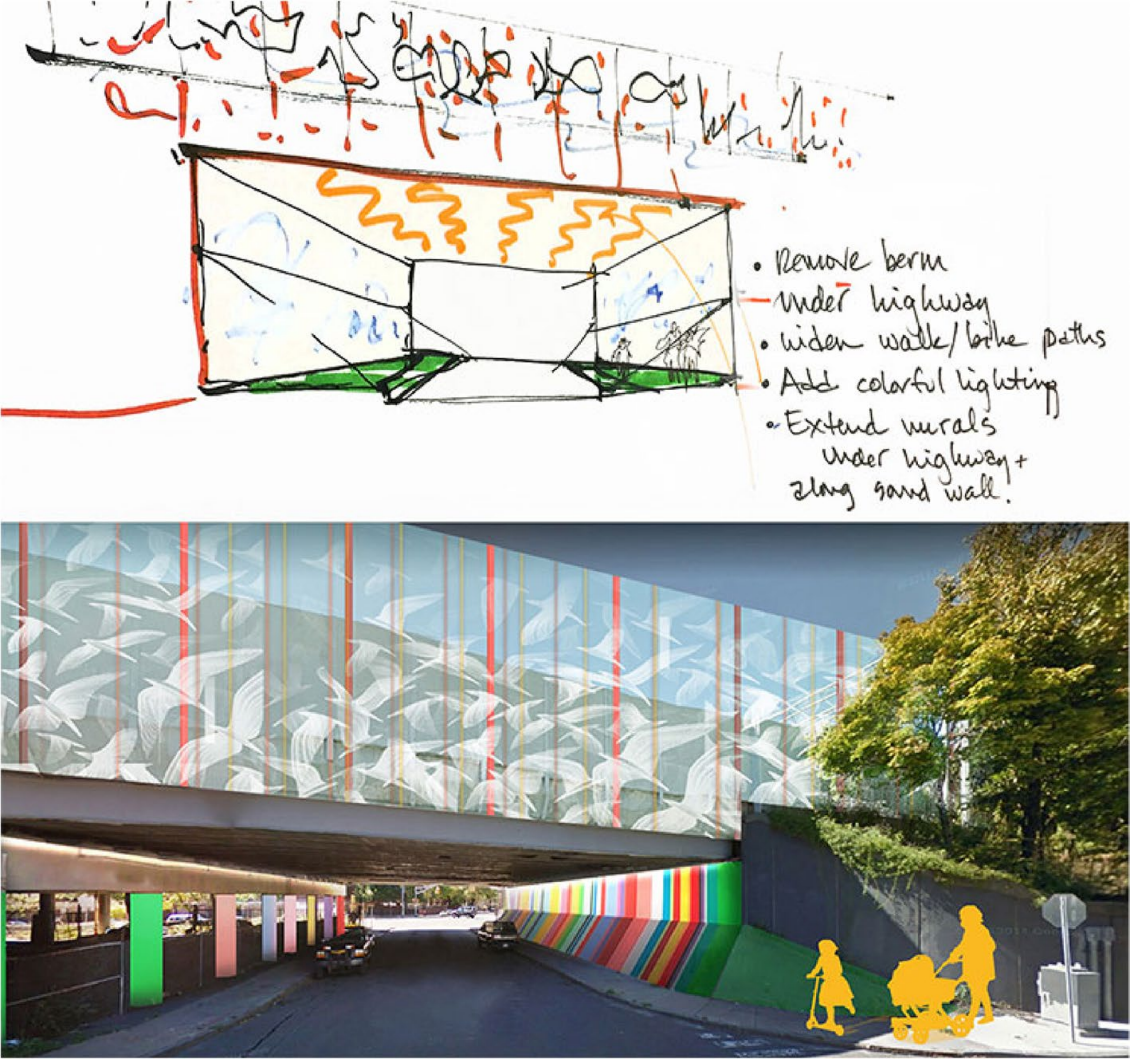
Proposed I-93 sound barrier above pedestrian underpass [34].

Listening sessions, which were followed by open houses which culminated with a day-long design charrette, allowed the partnership to catalyze broad community dialogue focused on TRAP [44]. During the Somerville planning charrette community members worked with architects and designers to reimagine areas along the highway. They generated potential solutions that were sketched in real time as seen in Fig. 2*,* which was also published in a public health post [34]. In addition to sound barriers, building level remediation was explored across groups.

In Somerville, the charrette was followed by biweekly community action and planning meetings convened by community partners. Key informant interviews indicate that Somerville partners were using several distinct strategies reaching far beyond noise barriers to mitigate the deleterious health effects of TRAP. These efforts began with resident engagement and education to build the base needed to advance change at the municipal and state levels.

#### Engagement, awareness and partnerships

Resident engagement and awareness were an ongoing focus for Somerville partners leading up to and following the HLA.

*So, it’s an invisible problem that most people don’t even recognize, and you know they see it doesn’t impact housing values at all, so apparently, so nobody really seems to care that much.*

Resident engagement and awareness strategies were seen as an important lever for advancing municipal and legislative action needed to alter the built environment for TRAP mitigation. Strategies to advance resident engagement described by key informants included increasing emphasis on multilingual materials and simultaneous interpretation at meetings as well as the inclusion of action steps that residents could take. Participants also described the importance of continued collaboration with immigrant serving organizations and grassroots organizers.

*…we do door to door work [outreach and education], literally just going through the you know the housing development, slipping flyers underneath their door for meetings. You know, we go into our English classes and talk to people directly about the work we’re doing. We flyer … different communities and invite people to these meetings where we know...where we try to put together a robust dialogue and then we try to facilitate conversations in a way that’s fair, so the loudest voice is not the only one that’s being heard, but all voices are being heard in these subject matters.*

**Fig. 3:**
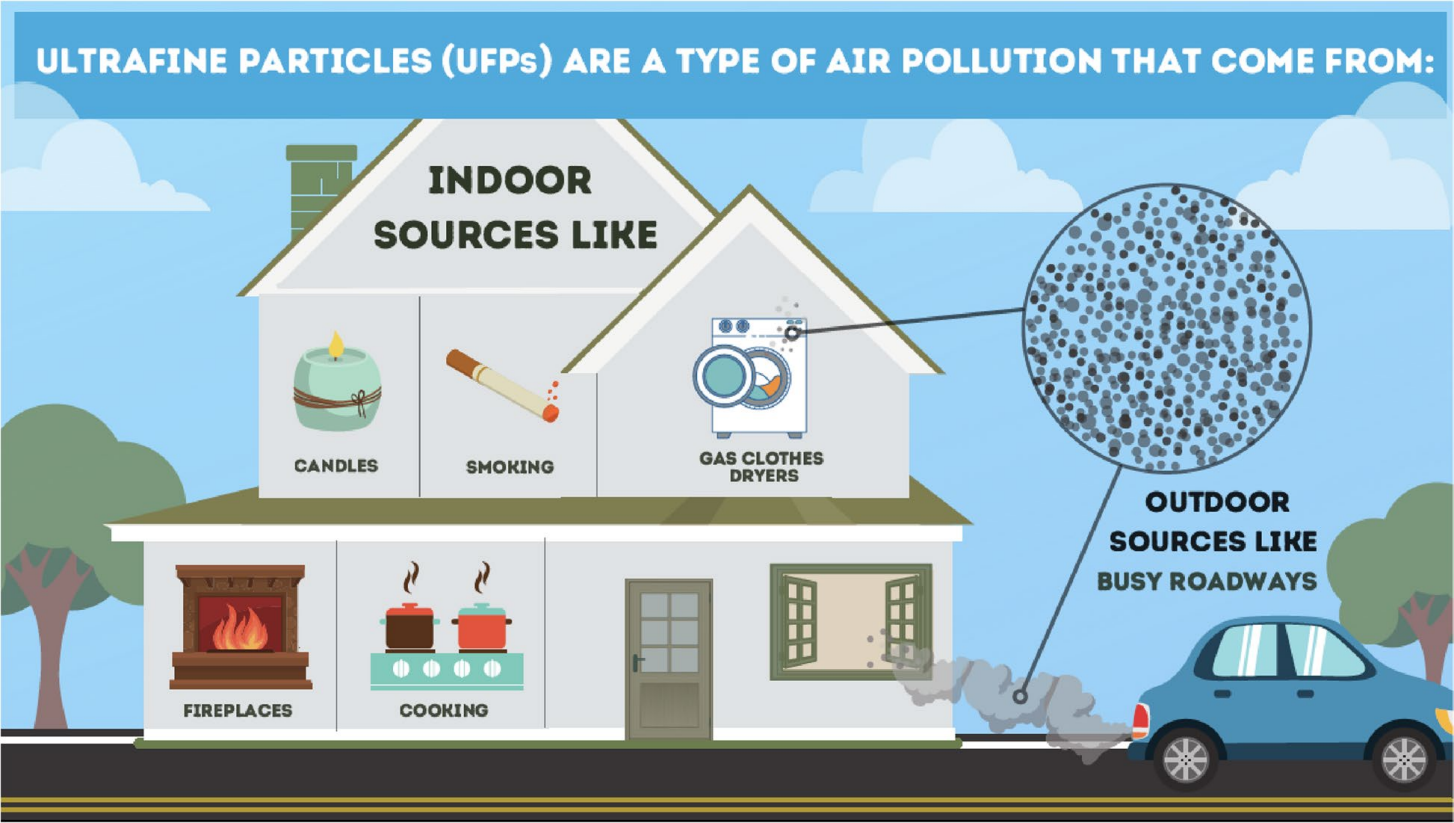
English version of TRAP Infographic

These strategies facilitated the engagement of diverse community residents in Somerville, which was a priority for the team because early on there was translation available at these open house events, but it was barely used, as open houses were attended predominantly by older, white, English-speaking residents. As such, the team engaged in conversations with immigrant communities facilitated by conversations with The Welcome Project, who hosted meeting through English classes and at housing health and resources fairs. These efforts prompted the project to develop infographics which further broadened the teams reach through the dissemination of the science in a way that was understandable to people with little science knowledge or not interested in spending time to figure it out as seen in Fig. 3.

The Somerville community partners approached the post-HLA period (that extended beyond the funded research) by partnering with other coalitions and groups with aligned values. An example of this was an effort to partner with another local environmental justice coalition that was working to advance change at the state level through legislative efforts.

#### Municipal level intervention

At the municipal level, Somerville organizing efforts focused directly on the built environment. This included contributing to discussions about the redesign of Foss Park (located near the highway an along a major roadway), discouraging residential housing and public parks near highways, and actively bringing air pollution mitigation strategies into efforts seeking to include bicycle and pedestrian safety improvements as part the Interstate-93 viaduct restoration project. In addition, there have been efforts for updates and improvements to the zoning regulations, which initially began with funding from the Kresge Foundation.

…*now we’ve been focused on Foss Park. Partly because it’s one area that there seems to be some desires by the state to transfer ownership, is not the right word, management of the park to the city, and that allows the city to have some leverage to say ‘well if we’re going to take it over. You know, we would like to see a remodel of the park and here are things we would like,’ so it, you know so we’ve been using a lot of the information [data] to guide that work.*

*One of the focus areas of Foss Park …, it became known that this group is interested in Foss Park and I mean [Somerville partner] is involved in all of this anyways, but when they needed a facilitator for the Community-led meeting that was trying to put together a petition to DCR [Department of Conservation and Recreation] related … Foss Park, [Somerville community partner] was asked to be one of the co-facilitators. Ultimately, the petition did include, within a long list of other things that the Community wanted…, made reference to the Charrette report, and spoke to the desire that whatever the final outcome of the Foss Park redesign that it’s reflective of air pollution mitigation strategies….*

Earlier in their partnership, prior to the research and action study, Somerville partners, with funding from the Kresge Foundations, presented data to educate elected officials as well as municipal leaders from planning, community development and public health departments. Efforts included one on one meetings, the provision of testimony and letter writing focused on specific zoning amendments. Key informants from the city agencies reported that these efforts contributed to increased communication and coordination between agencies.

*I would also say that up until … three or four years ago, there were people on the planning board who really could have cared less [about] and were not interested in what we were doing. Even people who live near the highway, I mean it was very difficult [back then]. …we would show up and they totally did not want us to be there.*

*… I would say it was [name of Somerville partner]. I wouldn’t be able to pin down exactly the night that he showed up for the first time and started educating all of us in...so when you testify at a public hearing and several you get two minutes. And he’s probably...testified …14 to 15 times for two minutes and each one of those is like a new piece of evidence about why we need to care about this. In between those, I would have very long meetings with him, but I think he’s the reason that people are paying attention to this. At some point, he convinced us to be financially involved and partner with [university researchers], so you know...he convinced people some at some point …*

Indeed, continued efforts by Somerville partners overtime have changed their relationship to municipal level decision makers, who now see them as having critical information related to TRAP.

#### State level efforts

In Massachusetts, governance of the built environment occurs at multiple levels. Key informants described how efforts to address TRAP required state, in addition to municipal level, change efforts. As discussed, sound barriers along the highway requires state-level legislative action. More specifically, it requires engaging the Department of Transportation (DOT), which controls decisions related to sound barriers. However, when the DOT seemed impossible to influence, Somerville partners turned their attention to legislative advocacy, which was informed by the research but not supported by project funding.

Efforts to influence ventilation systems also ended up having state level targets for change. The building code in MA is governed at the state level, as such efforts to require minimum efficiency reporting value at level 16 (MERV 16) for ventilation systems would require changes to the state building code. Key informants described this as a challenge for municipalities, as it requires a home rule petition to make changes to the building code in a single town.

*I always kind of question what is the right jurisdiction for whatever we’re trying to achieve? Where does it live? And what we ended up discovering, but I think Massachusetts is kind of unique and compared to a lot of other places, because of how the building code works. Because we’re a Commonwealth it’s evenly applied across the state, whereas like New York and municipalities in California can adopt their own local building codes. Everybody’s treated equally in the eyes of the Commonwealth right, that’s kind of the MO of a Commonwealth. And there’s a rule in the enabling legislation for zoning that says that you cannot preempt the building code, so if the building code asserts jurisdiction over a topic or regulates something at all you can’t talk about it and zoning because that would be preempted into the building code… We attempted to require net-zero in zoning. We ended up doing a density bonus, but a lot of those things really belong in the Building Code…*

As a result, efforts were focused on education at the developer level. At the state level, Somerville community efforts, beyond the research, instead focused on environmental justice legislation and litigation.

*…[an area we were] successful, in at least one development, was having air filtration systems installed in the buildings at like MERV 16 or whatever number it is that’s the highest level possible. And we succeeded at that, and that’s kind of my goal is to have that be a requirement in every development close to a highway. …we’re running into state building code as an issue with that because we wanted to zone it, but they tell us we can’t …require it through zoning, it has to be building code and that’s a State issue.*

*… I’m interested in the effort to get MERV 16 air filtration in housing and perhaps in other buildings. We got it into a permitted lab building at Assembly Square [a near highway community]…*

*…we want to make sure, public housing, larger multifamily buildings, and commercial buildings where there’s, above a certain size, all within close proximity to highways, or class one, two, and three roadways that they are mandated to use these MERV [minimum efficiency reporting value] 16 filters. And that’s a big deal that we’re trying to link both the filtration piece to distance to this roadway infrastructure and then also we say for all new newly licensed or newly constructed buildings, daycare facilities, schools. etc that they also have to use MERV 16 filters, regardless of the distance to transportation infrastructure.*

Finally, Somerville partners had a long history of advocacy efforts to expand public transit. Although expansion of the Massachusetts Bay Transit Authority’s Green Line train was not the focus of the work reported here, Somerville partners saw these efforts as linked since both advanced environmental health.

*Well, the Green Line was a big part of that because, of course, if you, as soon as you start getting Green Line in that means that people are doing less driving....*

### Chinatown HLA

The Chinatown HLA was used to inform the neighborhood’s master planning processes. Listening sessions were held in the context of existing community meetings [41]. This involved municipal planning partners documenting community priorities at the intersection of public health and urban planning [41].

*…. in the Chinatown HLA process …we worked with a group of students from the Harvard healthy places class. … they did stakeholder interviews with older adults that [community research partner name]organized …, they did some systematic coding to identify the different places where residents saw health in the built environment and social environment intersecting in their lives. … they also did data collection so looking at secondary data sources and seeing…using those codes to guide what was the secondary data that they collected, so a lot about green space they then [used inventories] ...the Boston tree inventory and …they did some primary data collection of their own, … auditing open spaces … And they put that together in a report that organized it into these are the different pathways towards health that we heard coming up in [resident] conversations…while the students were doing all that great data collection and analysis we started building relationships with the master planning committee and …leadership groups in Chinatown …by the end of the time that the Master Plan came out, we were able to present it back to the master plan committee and sort of start having conversations …*

Six areas of public health concern were identified: housing, public realm, air quality, climate change, walkability, and open space [41]. Publicly available data was examined to further inform each of the priority areas. Solutions and recommendations were generated at a dynamic, day-long community design charette attended by ninety residents, along with regional planners and volunteers [35]. Consensus building was used during the charrette to develop broadly agreed upon solutions and recommendations [35].

The Chinatown HLA process resulted in the community coming together to develop a master plan that incorporated TRAP mitigation strategies. More specifically, for Chinatown, TRAP was not a stand-alone health priority, instead through community conversations and intentional planning, TRAP mitigation strategies were embedded in the broader community plan. For example, affordable housing was a priority and was paired with adequate filtration and ventilation.

*And I think our job as part of the [research] team was to balance air quality as part of it, and then also say like if you’re talking about affordable housing and you’re talking about open space, there is space to talk about air quality within that and it doesn’t have to be a conversation about air quality. But that there are opportunities to draw attention to the co-benefits of these strategies.*

Key informants similarly reported that these conversations emphasized the placement of centralized air system away from the highways onto the roofs and include higher grade filtration.

*we’re building the new Josiah Quincy Upper School, and during the design process, one of the issues I focused on is are we able to build a school knowing where the physical building will be located, and what steps could we do to make the building more compatible, I guess is the word, or, or, more environmentally friendly, knowing we’re dealing with the air pollution. So, the city of Boston public facilities team that oversees the construction of city buildings, working closely with the Boston School Department designed a building that would factor in the air pollution. And so, they made different structural changes that would make it easier for, you know, for students to breathe in clean air, making sure that our water system is cleaner, which also goes with clean air. So that was that was one issue, and our open spaces and parks…*

To that end, broad community awareness of the nuances of TRAP and combined with the HLA allowed planners and residents to think creatively about mitigating TRAP. Like key informants in Somerville, Chinatown key informants highlight resident engagement and participation, as well as the need for municipal and state level intervention strategies that were beyond the scope of the HLA.

*…anytime you’re talking about new construction [in Chinatown] there’s definitely conversation about what’s the appropriate HVAC, the MERV filtration that’s appropriate to mitigate against it, the ultrafine- the UF- UFP, right, the ultrafine particulates…in terms of the legislation that’s going to require more sophisticated advocacy, right, that’s going to definitely um, you know that’s at the level of getting lobbyists involved or, or you know, because you have to start drafting appropriate language for legislation, or you know ordinances, and so that’s a whole another level of organizing. But ultimately, yeah, you need to have, you need to have people show up…*

#### Resident engagement and participation

Chinatown partners also focused on public engagement and increasing community understanding of the impacts of TRAP. Efforts in Chinatown included eliminating scientific jargon and the development of clear messaging and accessible materials. In Chinatown, organizations and active residents created the materials and messaging. Meanwhile, efforts to engage residents also involved participatory arts-based methods that led to the creation of public art and community forums.

*I am an artist ….Most recently, I’ve been working on an art project called Washing, which is about the legacy of the highways in Chinatown, and … the impact on present-day residents, which of course includes a ton about air pollution, noise, traffic… one [goal] of this project,...is helping people understand their place in community planning or their power... really their agency, and I think this project...we’re really talking a lot about the history of Chinatown and how folks have reacted to and resisted the highways and … a goal that we have as an artist team is to help folks understand that they have power….they have the ability to mobilize and change the course of what happens to their neighborhoods, …it’s not something that people are just born knowing and with air pollution, …[the research is] really helping us understand air pollution …[I see] science and research and facts and then I see an art project. … how do we build empathy for what it really feels like to know UFPs …, what does it really feel like to be coughing all the time, or to see your daughter get nosebleeds or because you can’t open the windows, or like just to be living with all this dust and particulate matter. I think that function is not just empathy for empathy’s sake, but I hope that we can use it as a way to reach out to policymakers and to planners as well as for the residents. I think there’s a function of feeling heard and understood. And … to share a little bit about what folks can do to protect themselves to bring this topic of air pollution back into …the conversation that people are having about you know what do we want for our neighborhood? Because air pollution is invisible… and in the end, there are so many other competing priorities … so [we are] …making a little bit of space for this conversation.*

Key informants reported these activities were intergenerational and deepened community dialogues about the impacts of the highway on resident health.

*What’s interesting about what she did as a public art project she involved all these people in talking about the history and the impact of the highways, and so...it wasn’t just a health focused project...and it wasn’t just a historical project or just a social justice project. But it kind of brought all these different pieces together like how the highway, how urban renewal destroys people’s lives, the injustice, the health impact of living with the pollution, what it did to the neighborhood, and that feeling of community was all kind of rolled up into one and I thought that was really powerful.*

More information about the *Washing* exhibit can be found at: https://www.washingchinatown.com/the-installation.

#### Municipal level intervention

In Chinatown, there were limited conversations about changing building code or zoning with municipal staff. Community partners did organize one conversation to explore the possibility of influencing the Boston Planning and Development Agency Public review process known as “Article 80” or large project review to require that developments pursue the LEED air quality credit when applicable [42]. However, they were not able to gain traction. However, the focus on air pollution within the Chinatown Master Plan was intended to influence the Boston Planning and Development Agency planning study, which would inform other City of Boston transportation and land use decisions.

Chinatown has also engaged in several development related efforts. This has included outreach and education, emphasizing building in ways that are protective, such as high-quality filtration and other design features that reduce exposure, with particular focus on the positioning of buildings and air intake systems.

*…every iteration of the [school] building plan since, including our current building plan, accounts for the pollution. So, it implements the best practices. I’m not too sure how much our architect and designers knew about the ultra-fine particulates, but certainly after being acquainted with [researcher], and we’ve done charrettes together and so on, so all our design teams are now very familiar with [the TRAP] research. So one of the modifications was to make sure air intake was on the rooftop instead of conventionally choose it on the ground level, you know behind the building or something, but in this case, they decide to put it on the rooftop because that’s the furthest away from the pollution, it’s on the on the side of the building furthest away from the Mass Pike [interstate 90] and the envelope for the buildings is very tight, so that it minimizes direct, direct um... the Pike, of course, these days, with COVID, you start to decide which is worse is that you know worse to die from air pollution or from COVID, but nevertheless. Also, the HVAC will incorporate MERV 14 filtration; it’s not the maximum of 16 but certainly MERV I think 12 and above is considered quite good.*

Similarly, there was a focus on redesigning parks and pedestrian walkways to mitigate exposure, this involved the incorporation of trees to build a green barrier around the park.

*…we have Tai Tung Park, which is probably one of the smallest parks in the city, but the State also has the Reggie Wong Park… I’ve been working with the state in the city over the last three years and we’re going to make some changes at Reggie Wong Park…*

Participants described community development and collaborative strategies such as using data to educate city officials and to build relationships with municipal planners and officials as well as the Department of Conservation and Recreation to advance TRAP reduction related goals. There was also a sense that building relationships and sharing information would increase developer awareness, although there were also direct efforts to educate and influence developers. These efforts were focused on increasing developer use of MERV 16 filtration as well as rethinking building position.

*So, I think that there are different levels of mitigation one is just individual by making people more aware. Maybe the best place to go for a walk is not too close to the highway or that to time your exercise when it’s not a heavy traffic period to keep your windows closed at certain times. And then I think on a community planning level, what are things that can be done, and more and more largely out of the research that [this project] has done, people are talking about high-quality air filtration because it seems to be the strongest mitigation measure that we can take around you know, protecting people and in terms of indoor air. I think people kind of learned that through [this research] and it’s become something that we and ask about when development proposals are coming up.*

#### State level intervention

Chinatown community partners expressed an interest in requiring MERV 16 ventilation, which would require change to the building codes at the state level. As in Somerville, the strategy for advancing this goal would require advocacy and education beyond the scope of the HLAs, that encourages residents to apply pressure to the city and state.

### Cross-cutting successes and challenges

Partners in Somerville and Chinatown experienced significant success advancing community level change to address TRAP. Achieving broad stakeholder engagement and resident awareness was perhaps their greatest win. Resident and stakeholder education are critical for building the broad support that could inform legislative action and community change [43].

*Chinatown has established a network of kind of building captains are block captains, who are most who are the activists in each of these buildings that then do outreach to their neighbors. And they utilize that a lot around their civic engagement work, so I just used a lot of those existing networks, and if we wanted a core of a dozen people, then it would be easy to say oh well, let’s either bring the Chinatown Resident Association steering committee together or call up these different block captains.*

Despite successes, challenges persist. Sustained action and active conversations focused on TRAP reduction is a continued challenge for community-based organizations given the multiple efforts happening in communities and the limited funding support. In the case of Somerville, continuous education of local officials, municipal leadership and staff has led to TRAP being viewed as a priority. As noted by one municipal leader below.

*…and we definitely have dedicated staff resources to this, both from the solicitor’s office trying to understand the right place for this type of legislation and then myself just holding...this just became a topic that we discussed in the course of doing the zoning overhaul. So, over the course of years, occasionally, we would have an air quality meeting. But we would stay in touch with you know, everybody... [researchers and community research partners]. To make sure that we didn’t get lost, because there were so many topics that we’re trying to tackle.*

*[we] seem to be much more accepted and listened to by the planning board in Somerville where all new construction projects have to go and get approval, because we kept showing up and saying, look you got to put good ventilation systems in these buildings. And on the one hand, they were, we were a pain in the neck to them, but on the other hand they knew we were right*….

*...[overtime] we built that relationship, and they trusted us, [laughs] they trusted the science, not necessarily us, but they trusted the work that we’ve done and have been supportive in this effort… now we’re working together…*

In Boston Chinatown, there has also been increased awareness among municipal leaders and staff, which is largely due to broad neighborhood engagement in the master planning process. However, in the case of both Somerville and Chinatown staff turnover and elections present the constant challenge of having to reestablish ties and reeducate local officials on the severity of the health threat posed by TRAP.

Participants in both Chinatown and Somerville reported an increased awareness of TRAP has contributed to community level changes including MERV 16 filtration in buildings. This has led to recommendations on the placement of air handling systems and windows in both communities. In Somerville, it has also led to administrative policy changes and resources being allocated to window retrofitting in buildings along the highways, as replacement windows that are tighter and allow less outside air in. In addition, both Chinatown and Somerville have plans for parks to be redesigned in ways that would mitigate TRAP exposures. A summary of strategies and successes by community can be seen in Table 1.

**Table 1:** Strategies and successes by community approximately

Community	Level Change	Strategies for Change	Successes
Somerville	Resident Awareness and engagement	Collaborative strategies: outreach and education through partnerships with organizations and media	Resident engagement TRAP is a priority for municipal leaders and included in discussions related to park redesign and housing development Changes in developer practices related increased level of MERV filtration as the result of education Increased municipal coordination around Window retrofitting in near highway housing
	Municipal Policy	Collaborative and campaigning strategies: Partnerships and educational efforts designed to influence local planning and zoning. Educational partnerships and direct outreach to influence developer behavior	
	State Policy	Collaborative, Campaigning and contesting strategies: Attempts educate and partner with leaders followed policy advocacy and litigation	
Chinatown	Resident Awareness and engagement	Collaborative strategies: outreach and education through community organizing and arts-based initiatives	Resident engagement Engagement across Chinatown neighborhood Agencies Prioritized TRAP in the master planning process Park and school redevelopment took TRAP into account in the planning Changes in developer practices related increased level of MERV filtration as the result of education
	Municipal Policy	Collaborative and campaigning strategies: Partnerships and educational efforts designed to influence local planning, housing policy and zoning	

Although both communities have made tremendous inroads with respect to building support at the municipal and state levels, challenges persist. The main challenge community partners face is governance of the built environment, which cuts across multiple departments and divisions at the municipal, state, and federal levels. On the one hand, Somerville participants reported increased coordination and buy in across municipal units including planning, public health, and board of health, on the other, it was unclear at times who had decision making power related to recommended changes. As reported by a Somerville municipal leader:

*…after a lot of research, we discovered that there’s the State law that says that air quality, the jurisdiction over air quality actually rests with local boards of health. And so, working with our solicitor’s office we actually even let the board of health know because we were talking to them about near highway air pollution and they didn’t even they weren’t even aware that there that’s within their jurisdiction, I kind of don’t blame them they’re like a lot of boards … volunteers.*

In theory, local bylaws and ordinances could consider setting building performance metrics, instead of building code standards. For example, a building needs to achieve a specific percent reduction in particulate matter (PM_2.5_) from ambient levels (of note PM_2.5_ was not a consideration in our proposal, which focused on UFPs); however, how they achieve said performance metrics cannot be dictated. Somerville officials had reservations about performance-based metrics since they are not as strongly grounded in research, at the same time the board of health and the inspectional service department did not feel they had the training and capacity to monitor air quality in this way. Moreover, as previously noted, changes to the building code recommended at the local level were under the purview of the state as they required changes to the building code. Such changes were described as complicated because they require a home rule petition, which participants reported was “not a step to be taken lightly”.

## Discussion

We set out to explore how community research partners translate research to build support for public health action that reduces TRAP exposure, the systems they target for change and the strategies and tactics they employ. The literature indicates engaging community stakeholders and residents in the research process, from planning and implementation to dissemination, facilitates research translation [30, 45]. We found that action groups in both Somerville and Chinatown were effective in research translation as well as catalyzing local public health action to reduce TRAP. Interview data indicated communities used TRAP related research findings to improve environmental health, by first developing a shared understanding of TRAP among community leaders and policy makers. This is consistent with the literature, which indicates CBPR can increase community capacity for action by creating a shared understanding [46].

Participants described the ways in which research related knowledge was transferred from community partners to public officials and staff as well as to other community leaders and residents more broadly through dialogue, outreach, and action [47]. Similar community action efforts in California have led to: 1) an increase in community member voices in the decision-making process; 2) community partners informing local planning efforts and legislative action; and 3) the elevation of TRAP in local planning efforts [48].

Notably, the Chinatown and Somerville communities are targeting multiple systems to advance change. Targets include developers as well as municipal and state government departments. More specifically, efforts are underway to shift developer practices directly as well as to change guidelines that govern building practices. Because these practices are governed by both state and municipal government, Somerville efforts have focused on both levels. Chinatown, meanwhile, has focused on developers and municipal government leaders. These identified targets are consistent with previous TRAP research which indicates educating developers and government leaders is a critical step in advancing efforts to mitigate the negative health effects associated with TRAP [49, 50].

Collaborative and campaigning strategies were most frequently referenced by participants when describing efforts in their respective communities. Collaborative strategies are appropriate when the target systems for change are open to and aligned with the change, whereas campaigning strategies are better suited to target systems that are willing to communicate but have not reached consensus toward alignment on a proposed change effort [51]. In the case of Somerville and Chinatown, participants described a growing understanding of TRAP and support efforts to reduce TRAP exposure, which left systems targeted for change open to discussions and, in some cases, aligned with proposed solutions.

Somerville informants described a history of past contesting and campaigning which led to increased awareness and support for current efforts. Specific tactics described by participants in both communities included capacity building, education, mobilization, persuasion and informing decision makers about science. Chinatown participants described using the arts and storytelling, which have a long history of inclusion in community action efforts [52, 53]. In both communities, we found the involvement of a regional planning partner critical for both implementing and sustaining local efforts. This finding is consistent with the implementation science literature which indicates external facilitation can help action groups or individuals better understand and strategize what is needed for change to happen and how to successfully implement a given innovation [54–57].

This research is not without limitations. We interviewed community members in both communities who have been involved in TRAP related action efforts. Because this work has been going on for many years, in some cases participants described efforts associated with earlier grants. Community and academic investigators on the steering committee were able to distinguish between efforts, but for their targets and those involved in only community public health action, the different projects that have occurred over time blended together, making it hard to distinguish which data was specific to the most recently funded research and action study. In addition, although we were able to speak with multiple actors from each of the communities, we were not able to engage state leaders in the interviews, limiting our understanding of how state leaders perceive local efforts. Nonetheless, this research is important in that it describes local efforts to translate environmental science into public health action.

## Conclusions

CBPR is an essential tool for identifying priorities for reducing TRAP and relevant interventions in communities. We partnered with neighboring communities; however, each took a very different approach to addressing TRAP. The research to action mechanism, supports CBPR and affords communities the ability to advance TRAP reduction efforts in a way that is locally meaningful, leveraging their respective strengths. External facilitation was key to sustaining momentum in both communities.

## Data Availability

More information about the CAFEH study and available data reports can be found at: https://www.cafehresearch.org/. Please contact Linda Sprague Martinez spraguemartinez@uchc.edu to request the data from this study.
